# Marine Oil Spill Detection from SAR Images Based on Attention U-Net Model Using Polarimetric and Wind Speed Information

**DOI:** 10.3390/ijerph191912315

**Published:** 2022-09-28

**Authors:** Yan Chen, Zhilong Wang

**Affiliations:** 1 China JIKAN Research Institute of Engineering Investigations and Design, Co., Ltd., Xi’an 710043, China; 2 School of Resources and Environmental Engineering, Anhui University, Hefei 230601, China

**Keywords:** marine oil pollution, oil-spill detection, synthetic aperture radar, deep learning

## Abstract

With the rapid development of marine trade, marine oil pollution is becoming increasingly severe, which can exert damage to the health of the marine environment. Therefore, detection of marine oil spills is important for effectively starting the oil-spill cleaning process and the protection of the marine environment. The polarimetric synthetic aperture radar (PolSAR) technique has been applied to the detection of marine oil spills in recent years. However, most current studies still focus on using the simple intensity or amplitude information of SAR data and the detection results are not reliable enough. This paper presents a deep-learning-based method to detect oil spills on the marine surface from Sentinel-1 PolSAR satellite images. Specifically, attention gates are added to the U-Net network architecture, which ensures that the model focuses more on feature extraction. In the training process of the model, sufficient Sentinel-1 PolSAR images are selected as sample data. The polarimetric information from the PolSAR dataset and the wind-speed information of the marine surface are both taken into account when training the model and detecting oil spills. The experimental results show that the proposed method achieves better performance than the traditional methods, and taking into account both the polarimetric and wind-speed information, can indeed improve the oil-spill detection results. In addition, the model shows pleasing performance in capturing the fine details of the boundaries of the oil-spill patches.

## 1. Introduction

The ocean not only provides the habitat for marine biology on earth, but also provides resources for human beings [1]. With the rapid development of marine trade, marine oil pollution is becoming increasingly severe. Statistically, 5.86 million tons of oil have leaked from seaborne vessels alone in the past 50 years [2]. There is a large amount of toxic compounds and heavy metals in crude oil, which will rapidly enter the marine ecological cycle through the food chain and damage the health of the marine ecosystem once an oil spill occurs [3,4]. Therefore, detection of marine oil spills is important for effectively starting the oil-spill cleaning process and the protection of the marine environment.

The rapid development of satellite remote-sensing technique has provided us with new research means for marine oil-spill detection [5,6,7,8]. Currently, synthetic aperture radar (SAR) has become the primary method of oil-spill detection, because of its all-day, all-weather, large-scale, and high-resolution imaging capabilities [9,10]. SAR satellite remote-sensing images with wide coverage allow for long-term and continuous monitoring of oil spills in the ocean.

In general, oil-spill patches on the sea surface exhibit dark spots and the boundary between the oil-spill patch and the surrounding water is quite visible in SAR images, due to the fact that the oil film can attenuate the Bragg waves on the ocean surface [11]. Based on the above phenomenon, the traditional methods of oil-spill identification with SAR images typically concentrate on studying the grayscale and the texture characteristics of sea-surface oil spills in SAR images. For example, Topouzelis et al. [12] proposed a decision-tree-forest method to detect oil spills in SAR images based on the intensity value of the image and the texture traits. Xu et al. [13] presented a method to detect oil spills on RADARSAT-1 image using the amplitude information of the images. However, besides the oil spills, some other look-alikes, such as low wind-speed regions, red tides, and the leeward side of islands could also show backscattering characteristics [14,15], which means that the traditional methods can hardly distinguish the oil-spill patches from the look-alikes by only considering the intensity or amplitude value of the image. Therefore, in the last decade, researchers have studied the topic of detecting oil spills in SAR images using more polarimetric information. The work by Migliaccio et al. [16] revealed that the copolarized phase difference can be utilized to effectively recognize oil spills in Sentinel-1 SAR images, and even some look-alikes existed in the images. In [17], polarimetric decomposition parameters (entropy, mean scattering angle, and anisotropy) were introduced into the oil-spill detection algorithm, and the results showed that the polarimetric features were helpful for effectively distinguishing oil spills, seawater, and most other look-alikes. In addition, some researchers have shown that environmental information, such as wind speed, can help distinguish between oil spills and look-alikes, so some studies have utilized wind-speed information in oil-spill detection. Frate et al. [18] presented a method to enhance oil-spill recognition systems using wind-history information. Naz et al. [19] used remote-sensing images and wind-speed data to model the oil-spill weathering process, which fully proved the critical role of wind speed in distinguishing oil spills.

For the past few years, the rapid development of artificial-intelligence technology (especially deep-learning methods) in oil-spill detection using SAR images has attracted attention. These recent studies have shown that deep neural networks have a remarkable ability for nonlinear expression and massive data mining [20,21] and can significantly improve the accuracy of marine oil-spill detection [22,23]. Garcia et al. [24] developed an algorithm for oil-spill detection from SAR data using a combination of two neural networks. In [25], a convolutional network with 12 weight layers used for oil-spill detection from spaceborne SAR images was designed, but only the amplitude information of SAR images was taken as the input data of the model. Similarly, only taking the amplitude as the input, Krestenitis et al. [22] demonstrated the good performances of a deep convolutional neural network in oil-spill detection with regard to the traditional method using a Sentinel-1 SAR dataset.

To sum up, although more and more studies have tried to use deep-learning-based methods to detect oil spills from PolSAR images and showed promising performance, very few of them have considered both the rich polarimetric information of PolSAR data and the weather condition of the sea surface, and most current studies still focus on using the simple intensity or amplitude information. This leads to the problem that most current oil-detection algorithms can hardly distinguish oil spills from look-alikes, and the profiles of the boundaries between oil films and water cannot be effectively retained. Based on this, this paper proposes a new PolSAR oil-spill-detection model. In this model, an attention U-Net architecture is employed, which is helpful to focus more on feature extraction and better distinguish oil films from look-alikes. Besides, both the rich polarimetric information of PolSAR data and the wind-speed information of the sea surface are utilized when training the model.

The rest of this paper is organized as follows. Section 2 introduces the basic information of the PolSAR dataset used for oil-spill detection and presents the Attention U-Net oil-spill-detection (AUOSD) method; then, the experimental results are described in Section 3; finally, we draw conclusions in Section 4.

## 2. Materials and Methods

### 2.1. PolSAR Oil-Spill-Detection Dataset

The PolSAR oil-spill-detection dataset should be collected before setting up the detection model. In addition, the number of images in the dataset should be sufficient, so that a robust deep-learning model can be obtained. In this study, we used images acquired by the Sentinel-1 satellites to detect oil spills (the basic details of the Sentinel-1 imagery are listed in Table 1). It is essential to point out that the Sentinel-1 data have a high temporal resolution and can be freely downloaded (https://scihub.copernicus.eu/, accessed on 10 July 2022). Hence, they provided the required data support for us to build a data-driven deep-learning model. As has been concluded in some previous studies [26], SAR imagery is more suitable for oil-spill detection under a medium incidence angle (20–45°). It can be seen in Table 1 that the incidence angle of the Sentinel-1 dataset is in this range. Therefore, the Sentinel-1 images used in this study are generally suitable for detecting oil spills.

**Figure 1:**
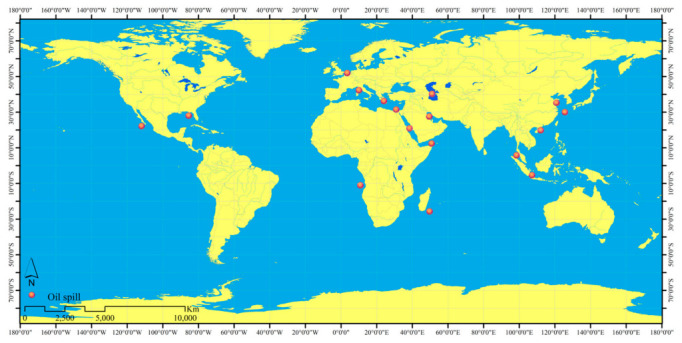
The location information of the oil-spill events used for model training.

To improve the interpretation ability of a deep-learning-based method, the dataset used for training should be sufficient and widely representative. In this study, we acquired and processed 35 Sentinel-1 images provided by the work in [17] (the locations of oil spill events as shown in Figure 1, and more detailed information is provided in Table 2) to construct a dataset for training the attention U-Net model of oil-spill detection. In the data-pre-processing steps, geocoding and speckle reduction [27] were undertaken.

For dual-polarimetric SAR images, the backscattering signal of each pixel contains both amplitude and phase information, which can be represented by a 2 × 2 complex scattering matrix *S* [28]:

(1) $$S=\left[\begin{array}{cc} 0 & 0\\ S_{\mathrm{VH}} & S_{\mathrm{VV}} \end{array}\right]$$

with

(2) $$S_{\mathrm{VH}}=\left|S_{\mathrm{VH}}\right|e^{\varphi_{\mathrm{VH}}i}$$

where the *S*_VH_ term is the cross-polarization channel, and the *S*_VV_ term is the co-polarization channel. $\left|S_{\mathrm{VH}}\right|$ denotes the amplitude, $\varphi_{\mathrm{VH}}$ represents the phase, and i is the imaginary unit. Then, the corresponding target-scattering vector *k* can be expressed as:

(3) $$k={\left(\sqrt{2}S_{\mathrm{VH}},\;S_{\mathrm{VV}}\right)}^{T}$$

The Sentinel-1 dual-polarimetric SAR data can also be represented by the following polarimetric covariance matrix:

(4) $$C_{2\times 2}=kk^{Y}=\left[\begin{array}{cc} C_{11} & C_{12}\\ C_{21} & C_{22} \end{array}\right]=\left[\begin{array}{cc} 2{\left|S_{\mathrm{VH}}\right|}^{2} & \sqrt{2}S_{\mathrm{VH}}S_{\mathrm{VV}}^{*}\\ \sqrt{2}S_{\mathrm{VV}}S_{\mathrm{VH}}^{*} & {\left|S_{\mathrm{VV}}\right|}^{2} \end{array}\right]$$

where *Y* represents the conjugate transpose, and * is the conjugate operator.

The polarimetric-target-decomposition technique plays an important role in target detection, classification, and geophysical-parameter retrieval [29,30], and can reflect the scattering mechanisms of the targets well. In order to make full use of the polarimetric information of Sentinel-1 images in oil-spill detection, we obtained the polarimetric-decomposition parameters (the polarimetric entropy (*H*), polarimetric anisotropy (*A*), and mean scattering angle (*α*)) from the Sentinel-1 dual-polarimetric data by adopting H/A/Alpha decomposition [31].

The decomposition parameter *H* describes the disorder of the various different scattering types (i.e., the randomness of the scattering). The value of *H* is between 0 and 1, and a larger *H* value represents a more chaotic scattering trait. Although *H* is an effective scalar feature to describe the randomness of scattering problems, it cannot completely describe the ratio relation of eigenvalues of the polarimetric-covariance matrix, and hence parameter *A* is a good supplement. The value of *A* is between 0 and 1, and a very low value means that there is only one primary type of scattering in the region. *α* is the key parameter used to identify the main scattering mechanism, with a value between 0 and 90. A lower α value indicates that the main scattering mechanism is single scattering, and a medium or higher α value denotes that the main scattering mechanism is double scattering.

Besides the polarimetric characteristic of SAR data, taking into account the marine environmental conditions, such as the wind-speed information, can also help to improve the accuracy of oil-spill detection. The research by Skrunes et al. [32] showed that the scattering trait of the sea surface varies in different wind-speed conditions. Therefore, to improve the feature-discrimination capability of the proposed model, the wind-speed information is also input into the designed model. In this study, we obtained the wind-speed information by the use of SNAP 8.0 software. The wind speed was estimated using the C-band Model 5 (CMOD5) model developed by Hersbach et al. [33] for C-band radar signals. Given the wind speed, the relationship between the normalized radar cross-section (NRCS) and the incidence angle in the CMOD5 model is:

(5) $$\sigma_{VV}^{0}=b_{0}{\left[1+b_{1}\cos\left(\emptyset \right)+b_{2}\cos\left(2\emptyset \right)\right]}^{1.6}$$

where $\sigma_{VV}^{0}$ represents the NRCS of the *VV* polarization and ∅ is the incidence angle. $b_{0}$, $b_{1}$, and $b_{2}$ are the functions constructed by the wind speed and incidence angle.

Finally, taking the elements of the polarimetric covariance matrix in (4), the Cloude–Pottier polarimetric-decomposition parameters, and the wind-speed information as the input of the model, the feature set for oil-spill detection can be defined as a vector *γ*, which is:

(6) γ=[C11,C22,Re(C12),Im(C12),H,α,A,WS]

where Re and Im represent the real and imaginary parts, respectively. *WS* represents the wind-speed information. That is to say, along with other images (the original SAR image and the polarimetric-decomposition-parameter images), the wind-speed image is directly input into the proposed oil-spill-detection model.

### 2.2. The Attention U-Net Oil-Spill-Detection (AUOSD) Model

By inputting the feature vector *γ* of the training samples, the neural network-based oil-detection process is to train and find the parameter set θ of network $f_{\theta }(\cdot )$ that achieves the smallest sum of loss function between the detection results $f_{\theta }(\gamma (i))$ and the real sample labels a(i):

(7) $$\hat{\theta }=\arg\min_{\theta }\sum_{i}^{M}\left|f_{\theta }(\gamma (i))-a(i)\right|$$

where *M* is the number of training samples. Once the networks are well trained, then given a new feature vector γ’, the corresponding oil-spill-detection result can be deduced by:

(8) $$F=f_{\hat{\theta }}(\gamma ’)$$

**Figure 2:**
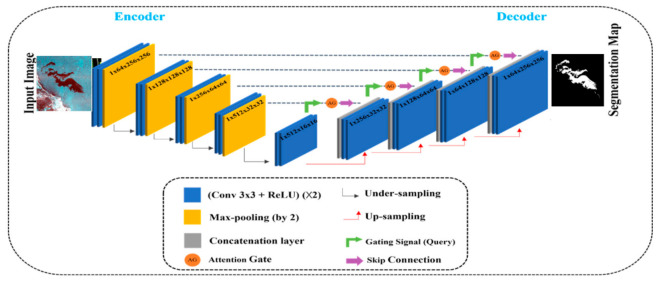
The architecture of the AUOSD model.

**Figure 3:**
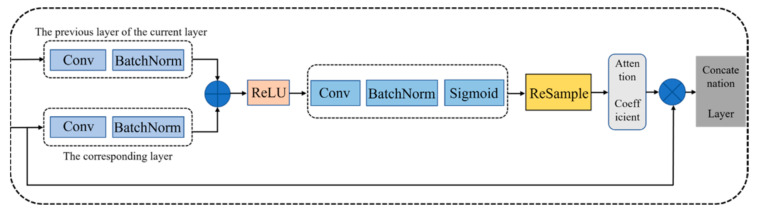
The internal AG modules in the AUOSD model.

The proposed AUOSD model adds attention gates (AGs) [34] to the U-Net network architecture [35], which ensures that the model focuses more on feature extraction. To better distinguish oil spills from the look-alikes, we used both polarimetric information and the wind speed as the input data. The structure of the proposed model is shown in Figure 2, which includes two main parts: (1) a contraction path and (2) a symmetric expanding path. The contraction path is the encoder part, in which two 3 × 3 convolutional layers are repeatedly applied to each layer, with each followed by a rectified linear unit (ReLU) and a 2 × 2 max-pooling operation with stride 2 for down-sampling. At each down-sampling step, the number of feature channels is doubled, and the feature information (such as the location and semantics of the image) is extracted. The symmetric expanding path is the decoder part and performs the up-sampling operation, where each module contains a concatenation layer and two 3 × 3 convolutional layers, to reduce the number of feature channels by half, with each convolutional layer followed by the same ReLU. In the meantime, the symmetric skip-connection structure connects the contraction path, the corresponding layer of the symmetric expanding path, and the AGs. In the up-sampling stage, the previous layer of the current layer and the corresponding layer are connected to the AGs. The concatenation layer is obtained after processing the feature information of the two layers inside the AG modules (Figure 3). The purpose of adding the AGs to U-Net is to focus the model’s attention on the feature targets in the image, thus improving the network’s ability to extract features in the receptive field [36].

In this study, to train the network, 400 samples with a patch size of 256 × 256 were selected. To prevent the over-fitting problem during training, data augmentation was used to increase the number of samples by both vertical and horizontal flipping, and the number of samples was then increased to 1200. We randomly divided the samples into three separate parts, namely, training, validation, and test parts, with a ratio of 5:2:3.

Neural-network models are often underfitted and overfitted during training, so the choice of hyperparameters is important to determine whether the model performs optimally and yields good results. In order to achieve the optimal configuration of the model hyperparameters during training, we performed a large number of experiments to adjust the settings of the different hyperparameters. We found that the optimal results were obtained when the learning rate was set to 0.001 and the batch size was set to 10 with the Adam optimizer. The hardware and software configurations for the experiments are listed in Table 3.

## 3. Results

### 3.1. Quantitative Assessment Indices

In order to objectively assess the performance of the different oil-spill-monitoring methods, several evaluation indices are introduced in this paper: the overall accuracy (OA), precision, recall, and F1-score.

The OA represents the ratio of the number of correct samples predicted by the model to the total samples, for which the formula is as follows:

$$\mathrm{OA}=\frac{TP+TN}{TP+FP+FN+TN}$$

where *TP*, *TN*, *FP*, and *FN* denote the number of true positive, true negative, false positive, and false negative samples, respectively.

Precision represents the proportion of correct samples in the model prediction of the various classification results, for which the formula is as follows:

$$\mathrm{Precision}=\frac{TP}{TP+FP}$$

Recall represents the proportion of correctly classified samples to the true value:

$$\mathrm{Recall}=\frac{TP}{TP+FN}$$

The F1-score is the harmonic average of the model precision and recall, which is given by:

$$F1=\frac{2\mathrm{Precision}\cdot \mathrm{Recall}}{\mathrm{Precision}+\mathrm{Recall}}$$

The ranges of the above four metrics are all between 0 and 1. The better the oil-spill-detection method performs, the larger the metrics are.

### 3.2. Oil-Spill-Detection Results

**Figure 4:**
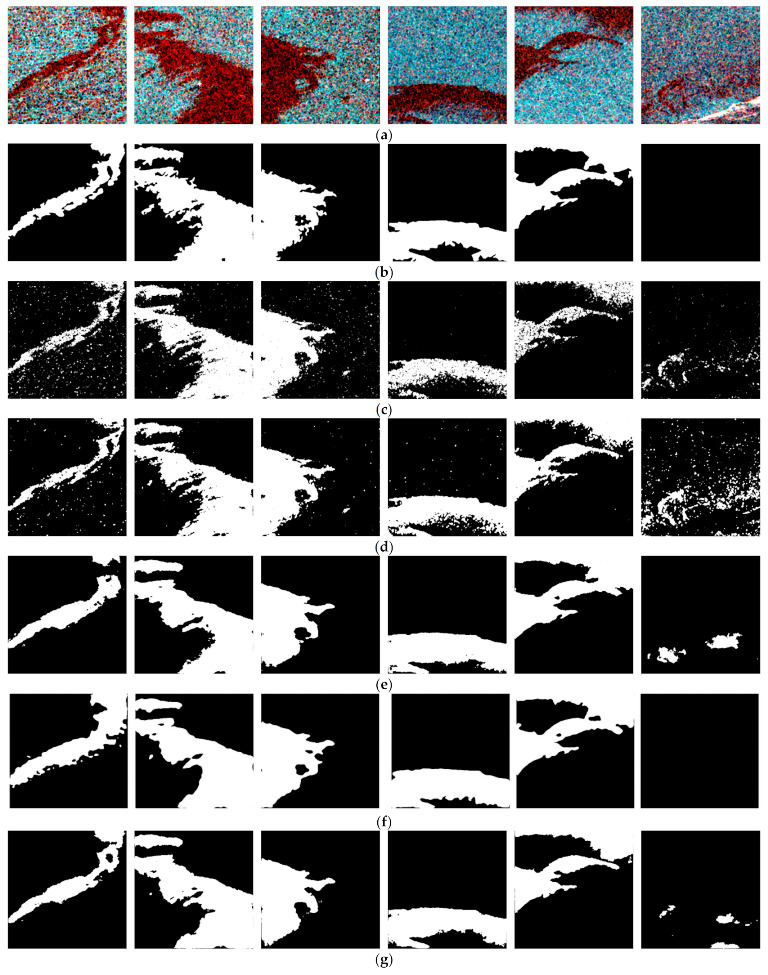
Oil-spill-detection results of the different methods: (**a**) the original Sentinel-1 PolSAR images; (**b**) the label images; (**c**) SVM; (**d**) Wishart; (**e**) U-Net model; (**f**) the AUOSD model without considering wind speed; (**g**) the proposed AUOSD model.

In order to fully verify the classification performance of the proposed model, the AUOSD model was compared with the traditional supervised PolSAR classification methods (Wishart, SVM) and the original U-Net segmentation algorithm, which also considers the polarimetric information and wind-speed information. The classification maps are shown in Figure 4, and the quantitative-evaluation results of the different methods are listed in Table 4. The image-interpretation time of each method is also displayed in Table 4.

Among the four methods, the Wishart and SVM classifiers obtained the lowest accuracies, with F1-scores below 90%. As can be observed in Figure 4, the speckle noise of the SAR images inevitably affected the traditional supervised-classification methods, despite a filtering approach being employed to suppress the noise. The detection results of the Wishart and SVM classifiers showed some sort of “salt–pepper” phenomenon and contained many spots with blurred boundaries, which seriously affected the accuracy of the classification. This indicates that the traditional methods only consider the low-level characteristics of the target and ignore the contextual information. In the meantime, there were more misclassification phenomena in the results of the traditional methods, especially for those objects that also had low back-scattering signals.

The deep-learning-based semantic segmentation algorithms took into account the contextual information of the imagery as well as the polarimetric information, which effectively reduced the influence of the speckle noise in the SAR images and improved the classification quality. Visually, the targets in the detection maps of the U-Net and attention U-Net model were more continuous, and the boundaries between the oil-spill patches and the water were clearer and more in line with the real situation.

As can be observed in Table 4, thanks to the employ of the attention gates, the oil-spill-detection performance of the proposed model was improved on the basis of the original U-Net model. The attention gates helped the model put more attention on the feature targets in the image, thus improving the network’s ability to extract features in the receptive field. From Figure 4, we can see that the fine details of the boundaries of the oil-spill patches were better retained by the AUOSD model; in addition, some misclassification problems that existed on the U-Net model-detection map were alleviated on the AUOSD model-detection map. In addition, to validate the necessity of integrating wind-speed information in oil-spill detection, the detection results by the AUOSD model without considering wind speed are also displayed in Table 4 and Figure 4. Clearly, when considering wind-speed information, the detection accuracy of the deep-learning model is improved to some degree.

## 4. Conclusions

Marine ecosystems offer substantial support and space for the sustainable development of human society, so the protection of marine ecosystems is of great significance. Marine oil-spill pollution has become a severe threat to the marine environment. Therefore, detection of marine oil spills is important for effectively starting the oil-spill cleaning process and the protection of the marine environment.

The development of the polarimetric SAR technique and deep-learning theory has provided us with new research means for marine oil-spill detection. Compared with the optical remote-sensing technique, the SAR technique can obtain information on the sea surface in all-day and in all-weather conditions. The typical traits of the oil-spill patches in PolSAR images are that they often exhibit dark spots and the boundary between the oil-spill patch and the surrounding water is quite visible.

However, the main challenge of oil-spill detection on PolSAR images is that some other look-alikes could also show backscattering characteristics. The existence of look-alikes exerts more difficulties when detecting oil spills. To better detect oil spills, especially from look-alikes, more information should be utilized. However, in many current studies, only the simple amplitude or intensity information is considered. Besides, since the back-scattering traits of oil spills and some look-alikes are quite similar, spatial semantic features (such as the object structures) needs to be fully exploited, which, is difficult for traditional detection methods.

Based on this, a deep-learning-based model for PolSAR oil-spill detection is presented in this paper. In this model, attention gates are added to the commonly used U-Net architecture, which is helpful for focusing more on feature extraction and better distinguishing oil film from look-alikes. To train the proposed model, sufficient PolSAR images were selected as the sample data. Then, both the rich polarimetric information of PolSAR data and the wind-speed information of the sea surface were utilized. The experimental results show that, even though the speckle-reduction approach was employed in the data-preprocessing step, many scatter points were still exhibited on the detection map of the traditional Wishart and SVM method. The experiments demonstrate the superiority of the proposed method, with high detection accuracy and good preservation of the boundary profiles of the oil-spill patches. It also validated that, by considering wind-speed information, the detection accuracy of the proposed model was improved to some degree. In addition, the introduction of the attention gates did indeed improve the performance of the U-Net model, especially in retaining the boundary profiles of the oil films.

Despite the promising performances, our work still encountered some problems that need to be studied in the further: (1) Big data is the foundation of constructing a deep convolutional neural network. In this study, 35 Sentinel-1 images with oil-spill patches were utilized to the train the network, which may not have been sufficient to ensure the excellent generalization ability of the proposed model. In addition, the polarimetric-scattering traits of oil films with different physicochemical properties are slightly diverse. Therefore, more sample datasets with different types of oil films should be considered in the future to ensure the robustness of the detection model. (2) This study only focused on the issue of oil-spill detection by dual-polarimetric SAR images. Nowadays, with the launch of more advanced satellites, people can get more and more quad-polarimetric SAR images. The task of oil-spill detection by quad-polarimetric data is much more complex, which will also be studied in our future work.

## Figures and Tables

**Table 1:** The basic details of the Sentinel-1 imagery.

Satellite Parameter	Description
Polarization	Dual (VV + VH)
Product type	Single look complex
Product level	Level-1
Mode	Interferometric wide
Band	C
Swath	250 km
Spatial resolution	5 m × 20 m
Incidence angle	33.86–42.93°

**Table 2:** Some detailed information of the oil-spill events.

Locations	Number of Oil-Spill Events	Ocurrence Time
Corsica Island, France	4	September 2017–Octorber 2018
Marseille, France	1	June 2017
Cabinda Harbor, Angola	3	May 2017–April 2019
Zeebrugge Harbor, Belgium	4	Octorber 2015–May 2017
Kharg Island, Iran	1	November 2019
Balilpapan Harbor, Indonesia	1	April 2018
Portsaid Harbor, Egypt	9	April 2015–April 2019
Jeddah Harbor, Saudi Arabia	6	May 2018–Octorber 2019
Khafji Harbor, Saudi Arabia	2	May 2017–August 2017
Baku, Azerbaidzhan	4	August 2019–January 2020

**Table 3:** The hardware and software configurations for the experiments.

Configuration	Version
CPU	AMD Ryzen 5 5600X 6-Core Processor
Memory	32 GB
GPU	NVIDIA GeForce RTX 3070
Language	Python 3.7
Programming	PyCharm 2020.3.4
Framework	PyTorch 1.6

**Table 4:** Quantitative-assessment results of the different oil-spill-detection methods.

	OA	Recall	Precision	F1	Processing Time (s)
SVM	0.9271	0.7421	0.9291	0.8251	18.4
Wishart	0.9369	0.8272	0.8927	0.8587	15.7
Unet	0.9556	0.8979	0.9095	0.9036	1.4
AUOSD (without considering wind speed)	0.9441	0.8702	0.9004	0.9011	1.5
AUOSD	0.9657	0.8962	0.9528	0.9236	1.5

## Data Availability

Not applicable.
